# Reconstruction of an Extensive Periocular and Bilamellar Defect of the Lower and Upper Eyelid Using Local, Regional and Free Chondral Graft Techniques: A Case Report

**DOI:** 10.29252/wjps.10.1.125

**Published:** 2021-01

**Authors:** Glenn De Weerdt, Pieter Luyten, Fabrice Dubrulle, Martain Loonen

**Affiliations:** 1 Department of Plastic, Reconstructive and Aesthetic surgery at AZ Groeninge Hospital Kortrijk, Belgium;; 2 Beverly Hills Sunset Surgery Center at Valiant Clinic Dubai, United Arab Emirates.

**Keywords:** Eyelid, Reconstruction, Chondral graft, Tenzel flap, Paramedian forehead flap

## Abstract

A 65-year-old female patient with a histologically confirmed basal cell carcinoma located at the right lateral lower eyelid was referred for surgical tumour excision and reconstruction of the periorbital area. The periocular zone was reconstructed in a two-staged procedure with bilamellar repair of both eyelids. An autologous chondral graft, mucosal advancement techniques and a periosteum-temporalis fascia hinged turnover flap were used for reconstruction of the posterior lamellae. A modified Tenzel flap and a paramedian forehead flap were used for reconstruction of the anterior lamellae. An acceptable functional and aesthetic outcome of the reconstruction was achieved.

## INTRODUCTION

Combined periocular and bilamellar eyelid reconstruction can be challenging due to the specific anatomic characteristic of this region and the need for both functional and aesthetic reconstruction. Many reconstructive techniques have been described to this day, but these usually focus on one specific region only.^1^^-^^3^ The level of reconstructive difficulty increases in case of extensive periocular defects. We present a challenging surgical case consisting of a full-thickness defect of the upper and lower eyelid and the periorbital areas after tumor excision. A creative combination of common and less frequent approaches was necessary in this case to reconstruct the defect. We successfully based our reconstruction on techniques originally described by Tenzel,^4^ Weinstein^5^ and Labat^6^ in combination with an autologous chondral graft and mucosal advancement techniques.

## CASE REPORT

**Fig. 1:**
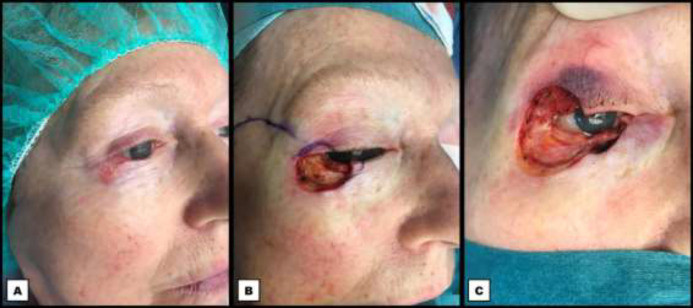
Right periocular zone prior to reconstruction. **A:** Preoperative photograph showing the basal cell carcinoma located laterally on the lower eyelid. **B:** Intraoperative photograph showing the periocular defect after the first tumour excision with positive section margins. A corneal protector is in place. **C:** Intraoperative photograph showing the periocular defect after the second tumour excision with negative section margins. Whilst passively elevating the upper eyelid, a full thickness defect of the lower and upper eyelid, and a defect of the lateral canthus is shown

A 65-year-old female patient was referred to our department by a dermatologist regarding a histologically proven superficial spreading basal cell carcinoma located at the right lateral lower eyelid with extensive tissue involvement. She had no significant medical history or comorbidities. Surgical excision of the lesion was carried out in two stages under general anesthesia: the first excision specimen still showed malignant cell nests in both lateral and medial section margins on histopathological examination. The second excision was effectuated five days later in a phased fashion until intraoperative frozen sections confirmed negative specimen edges. This resulted in a defect not only comprising of the anterior and posterior lamellae of the lower eyelid, which were already removed during the first stage, but also of the lateral canthus and the lateral upper eyelid including its lid margin. While awaiting the definitive pathology report, the wound was left open after each stage, protecting the eye with a tobramycine/dexamethasone ointment (Tobradex, Novartis Pharma NV, Vilvoorde, Belgium). The final histopathological examination confirmed the absence of tumour cells in the section margins, allowing reconstruction (Figure 1). 

The remaining periocular defect (about 40% of the upper eyelid, 75% of the lower eyelid, and the lateral canthus) was reconstructed through a two-staged procedure. Both eyelids were reconstructed separately as distinct aesthetic subunits in a bilamellar fashion. The first stage of the reconstruction took place twelve days after the first excision. To reconstruct the anterior lining of the upper eyelid a modified myocutaneous Tenzel flap was designed, superiorly based, in the lateral periorbital region. In comparison to the original technique described by Tenzel our flap was not semi-circular but angled, like a rhomboid flap.^4^

**Fig. 2:**
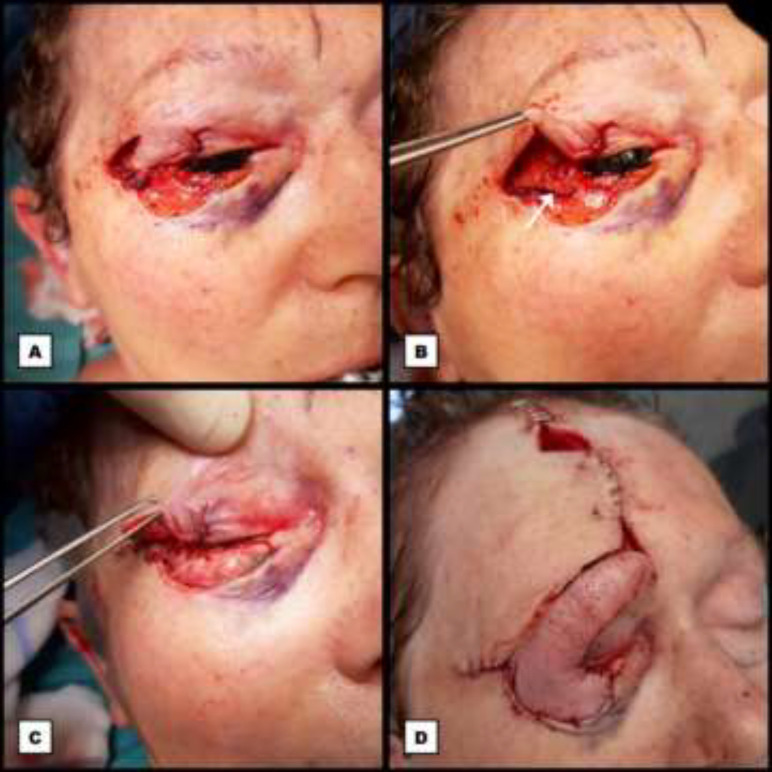
First stage of the periocular reconstruction. **A:** Intraoperative photograph showing the modified myocutaneous Tenzel flap advanced into the defect of the upper eyelid to reconstruct the anterior lamella. Posterior to this flap lie the superior component of the Y-shaped periosteal and temporal fascia sling, as well as the advanced palpebral mucosa which is sutured to the inferomedial border of the flap. **B: **Intraoperative photograph showing the inferior component of the medially hinged periosteal and temporal fascia sling (arrow) by elevating the modified Tenzel flap with forceps. **C:** Intraoperative photograph showing the autologous cartilage graft from the right concha sutured into the defect of the lower eyelid to reconstruct the posterior lamella. The modified Tenzel flap is elevated with forceps. Posterior to the cartilage graft lies the advanced palpebral mucosa which is sutured to the superior border of the graft. **D: **Intraoperative photograph showing the interpolated paramedian myocutaneous forehead flap transposed onto the cartilage graft to reconstruct the anterior lamella of the lower eyelid. Conventional closure of all donor sites with a small frontal defect remaining, left open to heal secondarily

Within this same region, a Y-shaped bifid flap from the periosteum and temporal fascia was created to reconstruct the lateral canthal ligament in order to restore the suspension of both eyelids. This Y-shaped turnover flap was hinged from lateral to medial as a sling to suture its superior component to the lateral edge of the remaining tarsal plate of the upper eyelid with an Ethilon® nylon 6-0 (Ethicon US, LLC) suture. A small palpebral conjunctiva advancement plasty was developed, which was able to reach the reconstructed upper eyelid margin, posteriorly covering the canthal sling. Then the myocutaneous Tenzel flap was advanced into the defect in de upper eyelid and was sutured subcuticular with antibacterial Monocryl Plus® poliglecaprone 25 5-0 (Ethicon US, LLC) and with Vicryl Rapide® polyglactin 910 6-0 (Ethicon US, LLC) sutures for the skin (Figure 2).

**Fig. 3:**
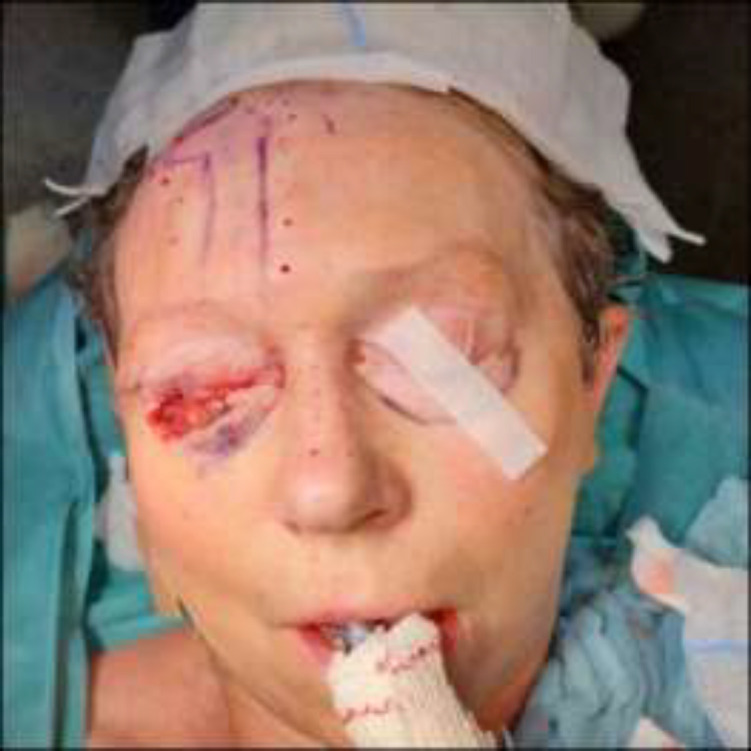
First stage of the periocular reconstruction with intraoperative marking of the forehead flap

Analogous to the upper eyelid, a minor palpebral conjunctiva advancement, was performed to posteriorly cover a chondral graft, which was added to reconstruct the posterior lamella of the lower eyelid. This chondral graft, with inclusion of the perichondrium to ensure ingrowth, was obtained from the concha of the right ear through a retroauricular incision. The graft was tailored to fit the defect and was sutured anterior from the conjunctiva into place with Ethilon® nylon 6-0 sutures to the lateral margin of the remaining tarsal plate of the lower eyelid and to the lateral orbital rim. Laterally, the inferior component of the Y-shaped hinged turnover flap was sutured to the cartilage as well with the same suture material, to provide suspension to the lower eyelid. In order to reconstruct the anterior lamella of the lower eyelid; an ipsilateral myocutaneous paramedian forehead flap was created, after design of a template (Figure 3). 

This flap, which remained vascularized via the right supratrochlear artery and contained a part of frontalis muscle, was rotated successfully into the remaining defect in the lower eyelid. To prevent devascularisation, no thinning of the flap was performed in this phase. It was sutured into place with Ethilon® nylon 6-0 (Figure 2). The lateral periorbital donor site, the frontal donor site and the auricular donor site were closed in a conventional manner, whilst leaving a tie over in the concha of the right ear in order to prevent seroma and hematoma formation. On the forehead, a small defect remained which was left open for secondary healing. All wounds were covered with nitrofural ointment (Furacine, Limacom, Diepenbeek, Belgium) and a grease gauze, except in close proximity to the eye where tobramycine/dexamethasone ointment was used. The later was also applied daily into the right eye (Fig. 2).

The second stage of the reconstruction took place one month after the first phase when the paramedian forehead flap showed adequate ingrowth (no signs of necrosis or venous congestion). Preoperative clamping of the pedicle was not performed. The second stage consisted of release of the pedicle of the forehead flap. The glabellar donor site was closed by a V-Y plasty, advancing a small remnant of the pedicle into the remaining donor site. The lateral part of the flap was tailored to the defect and was slightly thinned by resecting a portion of the subcutaneous tissue. In order to obtain a normal eyelid aperture, a partial lateral canthotomy was performed which released the cicatrisation that arose after the previous reconstructive stage. 

**Fig. 4:**
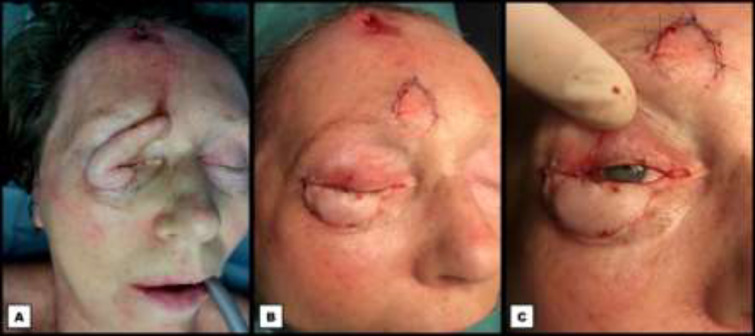
Second stage of the periocular reconstruction. **A:** Intraoperative photograph prior to release of the pedicle of the forehead flap showing an adequate flap ingrowth and a favourable healing of the donor site. **B:** Intraoperative photograph after release of the pedicle and lateral inset of the forehead flap after subcutaneous thinning and fixation to the lateral canthal ligament. The palpebral mucosa of the lower eyelid is advanced and sutured to the superolateral border of the flap. The frontal donor site is closed with a V-Y plasty. **C:** Intraoperative photography showing the passive aperture of the right eye after partial lateral canthotomy for scar release

The lateral part of the forehead flap was then fixed into the remaining defect with subcuticular Monocryl Plus® poliglecaprone 25 5-0 sutures. It was also sutured to the lateral canthal ligament with an Ethilon® nylon 6-0 suture to provide the flap with a favourable contour and suspension. The mucosa of the lower eyelid was then partially advanced to reach the new lateral eyelid margin. All wounds were further closed with Prolene® polypropylenesutures 6-0 (Ethicon US, LLC) sutures and were covered again with nitrofural or tobramycine/dexamethasone ointment and a grease gauze (Figure 4).

**Fig. 5:**
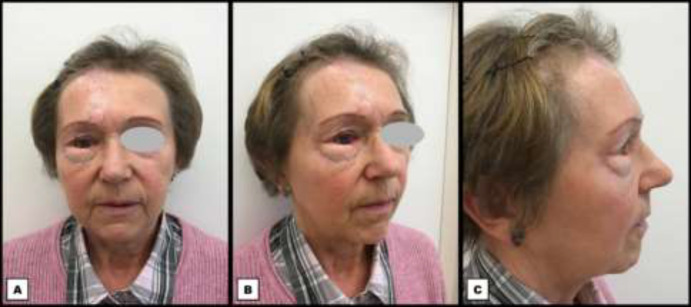
Postoperative photographs two months after the second stage of the periocular reconstruction. **A: **Frontal view. **B:** Oblique view. **C:** Lateral view

During the first weeks of follow-up, all flaps showed adequate ingrowth without any sings of necrosis. The remaining defect in the frontal donor site healed well secondarily. The reconstructed periocular zone healed well too, with acceptable results both functionally and aesthetically, and without changes in visual acuity. Two months after the second reconstructive stage, a minor ectropion of the lateral right lower eyelid was observed. This ectropion remained unchanged for the four next consecutive months. An ancillary corrective canthopexy was offered to the patient (Fig.5). Formal approval from the Ethical Committee of AZ Groeninge Hospital Kortrijk and written consent from the patient were obtained.

## DISCUSSION

**Figure 6:**
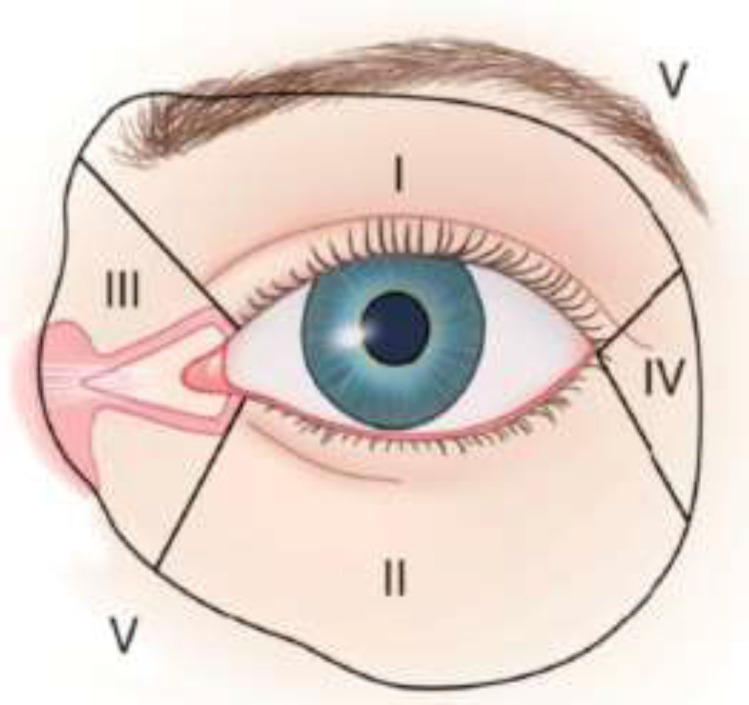
Periocular zones as described by Spinelli *et al*. **I**, Upper eyelid. **II**, Lower eyelid. **III**, Medial canthus. **IV**, Lateral canthus. **V**, Periocular contiguous area

Complete excision of the basal cell carcinoma in this case resulted in a full thickness defect in Spinelli’s periocular zones I and II, as well as in a defect in zone IV^1^ (Figure 6). Several reconstructive algorithms exist for reconstruction of full thickness defects of the eyelids. The algorithm presented by Fante *et al.*^7^ for full thickness defects of the lower eyelid suggests that defects <25% of the eyelid width can be closed directly, defects of 25-50% can be closed directly with lateral cantholysis, defects of 25-80% can be reconstructed with a tarsoconjuntival graft and coverage by a skin-muscle flaps, defects of 33-66% can be reconstructed with a semicircular flap (Tenzel flap^4^), defects of 50-75% can be reconstructed with a semicircular flap with a periosteal flap, and defects of 50-100% can be reconstructed with a tarsoconjuntival flap and a skin graft.

The algorithm for full thickness defects of the upper eyelid suggest that defects <25% of the eyelid width can be closed directly, defects of 25-50% can be closed directly with lateral cantholysis or with a tarsal rotation flap combined with skin-muscle flaps or a skin graft, defects of 25-75% can be reconstructed with a tarsoconjunctival flap and a skin-muscle flap, defects of 33-66% can be reconstructed with a semicircular flap (Tenzel flap^4^)with a periosteal flap, and defects of 50-100% can be reconstructed with a Cutler-Beard flap.^8^ The extent of the full-thickness defects in our case did not allow primary closure, not even with local tissue advancement. Also, the concomitant existence of these defects inhibited the use of several standard reconstructive techniques that rely on the ipsilateral unaffected eyelid as a donor site for free composite grafts or for local flaps (ex. Hughes’ tarsoconjunctival flap,^9^ Cutler-Beard lid-sharing flap^8^).

Flaps arising from the ipsilateral malar region (ex. Mustardé’s cheek advancement flap^10^) were not applicable either due to the extension of the defect in the lateral periorbital zone and the reduced elasticity of the patient’s tissues in this region. Since we had no intention of violating the integrity of the contralateral eyelids, harvesting free grafts form the patient’s left periocular region was not an option either.

The limitations in reconstructive options in this case led to the development of the two-staged approach with inclusion of a forehead flap. Since such a flap is rather bulky and the skin is much thicker, less pliable and of a different color, reconstruction of an eyelid with a forehead flap might seem undesirable. Nevertheless, this technique is sporadically used by other surgeons too, and is promoted for reconstructions of larger and more demanding periocular defects.^2^^,^^11^^-^^13^ The forehead flap has the advantage of having a reliable vascular supply, of being in close proximity to the periocular region and being easily harvested and rotated into the defect. In this case the forehead flap, as well as the other reconstructive techniques, contributed to an acceptable functional and aesthetic result.^2^^,^^11^^-^^13^

A search on Pubmed.gov (National Library of Medicine) showed other case reports of periocular reconstructions with a forehead flap. However, as in the reports of Price *et al*.^11^ and Elshamma *et al*.,^12^ many authors describe the use of this local flap for reconstruction of medially located eyelid defects or defects of the medial canthus. Modifications of this technique for periocular reconstruction, such as expanded pedicled forehead flaps,^14^ single-staged tunnelled forehead flaps,^15^ or forehead flaps with implantation of a chondral graft^16^ have been described too. Nevertheless, no comparable articles using the same combination of reconstructive techniques as described in this case report could be revealed by our Pubmed.gov search.

Alternative reconstructive techniques to reconstruct the periocular defect in this case might include a Fricke supraorbital adipocutaneous transposition flap^17^ for reconstruction of the anterior lamella of the upper eyelid and a lateral superior pedicled adipocutaneous transposition flap^18^ for reconstruction of the anterior lamella of the lower eyelid in combination with the autologous chondral graft, mucosal advancement techniques and a periosteum-temporalis fascia hinged turnover flap described in this case report. Another alternative for reconstruction of the anterior lamella of the upper eyelid might be a caudally pedicled frontalis muscle flap in combination with a full-thickness skin graft.^19^

Complete reconstruction of the upper and lower eyelids is overall known to be challenging and is often associated with a poor outcome due to complications such as graft necrosis, lagophthalmos, ptosis, lower eyelid retraction, and ectropion.^20^ Nevertheless, our case shows that a well anticipated and meticulously executed reconstruction can result in a functionally and aesthetically acceptable result after six months. The encountered complication of a minor lateral ectropion is one that can easily be addressed by a lateral canthopexy.

## CONCLUSION

Full-thickness defects of the upper and lower eyelids in association with a defect of the lateral canthus and respective ligament (Spinelli’s zones I, II and IV^(1)^) are challenging lesions to reconstruct. Nevertheless, our case report shows that such defects can be restored in an acceptable fashion trough a two-staged approach using a combination of a modified Tenzel flap, a paramedian forehead flap, a periosteal-temporal fascia hinged turnover flap, an autologous chondral graft, and mucosal advancement techniques. This is a minor lateral ectropion of the lower eyelid aside, without any complications.
